# Characterizing the Syphilis-Causing *Treponema pallidum* ssp. *pallidum* Proteome Using Complementary Mass Spectrometry

**DOI:** 10.1371/journal.pntd.0004988

**Published:** 2016-09-08

**Authors:** Kara K. Osbak, Simon Houston, Karen V. Lithgow, Conor J. Meehan, Michal Strouhal, David Šmajs, Caroline E. Cameron, Xaveer Van Ostade, Chris R. Kenyon, Geert A. Van Raemdonck

**Affiliations:** 1 HIV/STI Unit, Institute of Tropical Medicine, Antwerp, Belgium; 2 Department of Biochemistry and Microbiology, University of Victoria, Victoria, British Columbia, Canada; 3 Unit of Mycobacteriology, Institute of Tropical Medicine, Antwerp, Belgium; 4 Department of Biology, Faculty of Medicine, Masaryk University, Brno, Czech Republic; 5 Laboratory for Protein Science, Proteomics and Epigenetic Signaling (PPES) and Centre for Proteomics (CFP), University of Antwerp, Wilrijk, Belgium; 6 Division of Infectious Diseases and HIV Medicine, University of Cape Town, Cape Town, South Africa; Institut Pasteur, FRANCE

## Abstract

**Background:**

The spirochete bacterium *Treponema pallidum* ssp. *pallidum* is the etiological agent of syphilis, a chronic multistage disease. Little is known about the global *T*. *pallidum* proteome, therefore mass spectrometry studies are needed to bring insights into pathogenicity and protein expression profiles during infection.

**Methodology/Principal Findings:**

To better understand the *T*. *pallidum* proteome profile during infection, we studied *T*. *pallidum* ssp. *pallidum* DAL-1 strain bacteria isolated from rabbits using complementary mass spectrometry techniques, including multidimensional peptide separation and protein identification via matrix-assisted laser desorption ionization-time of flight (MALDI-TOF/TOF) and electrospray ionization (ESI-LTQ-Orbitrap) tandem mass spectrometry. A total of 6033 peptides were detected, corresponding to 557 unique *T*. *pallidum* proteins at a high level of confidence, representing 54% of the predicted proteome. A previous gel-based *T*. *pallidum* MS proteome study detected 58 of these proteins. One hundred fourteen of the detected proteins were previously annotated as hypothetical or uncharacterized proteins; this is the first account of 106 of these proteins at the protein level. Detected proteins were characterized according to their predicted biological function and localization; half were allocated into a wide range of functional categories. Proteins annotated as potential membrane proteins and proteins with unclear functional annotations were subjected to an additional bioinformatics pipeline analysis to facilitate further characterization. A total of 116 potential membrane proteins were identified, of which 16 have evidence supporting outer membrane localization. We found 8/12 proteins related to the paralogous *tpr* gene family: TprB, TprC/D, TprE, TprG, TprH, TprI and TprJ. Protein abundance was semi-quantified using label-free spectral counting methods. A low correlation (r = 0.26) was found between previous microarray signal data and protein abundance.

**Conclusions:**

This is the most comprehensive description of the global *T*. *pallidum* proteome to date. These data provide valuable insights into *in vivo T*. *pallidum* protein expression, paving the way for improved understanding of the pathogenicity of this enigmatic organism.

## Author Summary

Syphilis remains a major cause of morbidity and mortality worldwide. The bacterium causing syphilis, *Treponema pallidum* ssp. *pallidum*, has evolved into a highly distinctive organism that is only able survive (and be propagated) in mammals. In humans it can evade the immune system for decades with devastating consequences. Much remains to be learned about how it accomplishes this. Only a minority of its predicted proteins have been detected experimentally thus far. We aimed to more comprehensively characterize the proteins of this organism. Since it cannot be cultured *in vitro*, we cultured *T*. *pallidum* in rabbits and analyzed extracted proteins using different mass spectrometry methods, a manner of detecting proteins with high accuracy. In total, we detected more than half of the predicted number of proteins that could be expressed by this bacterium (N = 557). For approximately half of the proteins, we succeeded in characterizing their predicted cellular location using an array of bioinformatic tools and catalogued their function. This is the most comprehensive analysis of the *T*. *pallidum* proteome to date. This study lays the groundwork for other protein investigations of this unique organism.

## Introduction

*Treponema pallium* ssp. *pallidum*, henceforth referred to as *T*. *pallidum*, is the causative agent of syphilis, a multistage chronic disease with an estimated 8 million new cases per year [1]. Recent outbreaks of syphilis infection among certain populations such as men who have sex with men (MSM) [2], together with continuing substantial perinatal morbidity and mortality attributed to congenital syphilis infections [3], highlight the need for improved diagnostics and vaccine development.

*T*. *pallidum* is an obligate microaerophilic bacterial pathogen [4–6] that is aptly suited to invading mammalian tissue by the use of endoflagella that produce undulations in travelling planar waves [7], thereby driving its characteristic corkscrew-like movement [8]. The membrane of *T*. *pallidum* lacks lipopolysaccharide (LPS) and the loosely associated fragile outer membrane contains a low amount of proteins [8–11]. Many biomedical experimental approaches such as genetic manipulation have been hampered by its lack of *in vitro* cultivability [12]. Despite these limitations, numerous studies using *T*. *pallidum* harvested from the experimental rabbit model have increased our basic biological understanding of this unique organism, including the description of the genome [13,14], transcriptome [15] and proteome [16,17].

The *T*. *pallidum* Nichols strain genome was sequenced for the first time in the late 1990’s [13], revealing only 1041 predicted open reading frames (ORFs) on a 1.14 Mb circular chromosome, making it one of the smallest human pathogen genomes. Resequencing of the Nichols strain [14] identified 102 errors that were predicted to affect protein-coding genes and reduced the number of ORFs to 1039, 968 of which are predicted to be protein coding. Similar to other obligate pathogens such as *Mycoplasma pneumoniae* [18], *T*. *pallidum* is predicted to have lost many non-essential genes though genome reduction. This theory is supported by extensive genome-wide transcriptional analyses [15], which revealed the uniform expression of almost all *T*. *pallidum* genes during experimental rabbit infection. Consequently, *T*. *pallidum* has severely limited metabolic and biosynthetic capabilities, rendering it highly dependent on the host milieu and nutrients [19].

The predicted proteins found within *T*. *pallidum* range in size from 3,235 to 172,869 Da with an average size of 37,771 Da [13,20]. Early studies on *T*. *pallidum* polypeptides, including pre-MS analysis gel-based techniques and the use of recombinant DNA technology has been extensively reviewed by Norris *et al*. [17] and Schouls [21]. A large scale *T*. *pallidum* recombinant protein study included the construction of a bacterial artificial chromosome (BAC) library containing 901 of the 1039 *T*. *pallidum* predicted proteins for expression in *Escherichia coli* [22,23]; many of the expressed proteins were reactive with sera from syphilis-infected rabbits and/or humans [24,25] at different stages of infection as determined by serological reactivity studies. Subsequently, McGill *et al*. conducted a *T*. *pallidum* proteome investigation on *in vivo* expressed *T*. *pallidum* using gel-based approaches complemented with MALDI-TOF Mass Spectrometry (MS) and peptide mass fingerprinting [16]. A total of 88 polypeptides were identified and the immunoreactive potential of select proteins was characterized. Numerous bioinformatic approaches have been used to characterize *T*. *pallidum* proteins, including lipoprotein characterization [26], the determination of potential outer membrane proteins [27] and the re-annotation of *T*. *pallidum* strain SS14 hypothetical proteins [28]. However, despite rigorous analyses and major advances in genome sequencing, approximately 30% of *T*. *pallidum* proteins still have no known orthologues and at present cannot be assigned a biological function [13]. This ‘unknown’ category of proteins may represent an arsenal of genes encoding virulence factors specific for *T*. *pallidum* [20].

Progress has been made on understanding virulence and persistence strategies of this unique pathogen. Genetic sequence diversity is primarily localized in six hot spots [29] in *T*. *pallidum* ssp. *pallidum* and *T*. *pallidum* ssp. *pertenue* (the causative agents of Yaws), including regions encoding members of the paralogous *tpr* gene family consisting of 12 genes categorized into subfamilies I (*tpr*C, D, F and I), II (*tpr*E, G and J) and III (*tpr*A, B, H, K and L). The Tpr proteins contribute to antigenic variation that aids in immune evasion [30]. Nonreciprocal gene conversion occurs between donor sites and several variable regions (V1–7) in *tprK* [31] and these variable regions in the encoded protein are targets of the host humoral response during infection [32–35]. Host immune pressure is capable of selecting against certain TprK sequence epitopes [36] and TprK sequence variability can help evade the host immune response [35] during infection. Recombinant protein studies have confirmed surface exposure, bipartite architecture and porin function related to the outer membrane proteins Tpr C/D [37] and TprI [38]. Moreover, *T*. *pallidum* lipoproteins, of which Tp47 is the most widely studied [39–42], play an important role in immune system activation and evasion as reviewed by Kelesidis *et al*. [43].

With the recent evolution of robust highly sensitive tandem MS instrumentation, the comprehensive description of bacterial proteomes, also referred to as shotgun proteomics (reviewed by Semanjski *et al*. [44]), is achievable. Many current state-of-the-art proteomic studies have approached 80% coverage of the predicted expressed proteome [45,46]. The study of pathogens expressed *in vivo* is of particular interest since this would be the closest approximation of human pathophysiological conditions. For example, previous studies on the *Mycoplasma tuberculosis* proteome from guinea pig infected lungs during early and chronic stages of disease [47] have provided valuable insights into pathogen protein expression. However, interference of host proteins present in large excess can hinder MS detection of low abundance pathogen proteins. Thus, several strategies have been used to overcome issues of sample complexity to enrich bacteria cells, such as the use of density gradient centrifugation [16].

Using highly sensitive non-gel based complementary proteomic techniques, we sought to further elucidate the global proteome of *T*. *pallidum* in order to gain insights into the fundamental physiological state of *T*. *pallidum* during rabbit infection. Three biological replicates of *in vivo* cultured *T*. *pallidum* were subjected to multidimensional chromatographic separation and tandem MS/MS analysis whereby 557 *T*. *pallidum* proteins were identified at a high level of confidence, representing 54% of the predicted proteome. This is the first description of 499 *T*. *pallidum* proteins expressed *in vivo*, of which 106 were annotated as uncharacterized/hypothetical proteins. Detected proteins were comprehensively analysed to predict cellular localization and function. This unique ‘snapshot’ view of the *T*. *pallidum* proteome during infection extends our understanding of *T*. *pallidum* pathogenesis and forms the basis for further proteome investigations.

## Methods

### Rabbit inoculation and *T*. *pallidum* purification using Percoll density gradient centrifugation

Three biological samples, hereafter referred to as samples TPA-A, TPB-B and TPC-C, originated from three New Zealand White rabbits that were inoculated intra-testicularily with *T*. *pallidum* DAL-1 strain bacteria according to established methods [48]. Inoculations originated from two different bacterial stocks of DAL-1 strain bacteria, whereby sample TPB-B and TPC-C originated from the same stock. When peak orchitis was reached, on average 11–14 days post-inoculation, rabbits were sacrificed using T61 administration according to the manufacturer's instructions and the bacteria was extracted from the testes and purified using Percoll density gradient centrifugation as previously described [49]. Briefly, collected organisms were separated from host cellular gross debris by low-speed centrifugation at 34 800 g for 30 minutes followed by gradient separation via ultra-centrifugation at 100 000 g for 1 hour. Bacteria were quantified using darkfield microscopy and a counting chamber. For sample TPA-A, approximately 10^8−9^ treponemes were re-suspended and stored in 1 mL NaCl solution and frozen at -80°C. For samples TPB-B and TPC-C, approximately 10^8−9^ treponemes were re-suspended in 1 mL phosphate buffered saline (PBS) (HiMedia Laboratories, Mumbai, India) and frozen at -80°C. Two samples, TPA-A and TPB-B, were subjected to an extra thaw cycle before protein extraction due to inadvertent thawing during sample shipment. Each rabbit was tested serologically to rule out a naturally occurring infection with *T*. *paraluiscuniculi*.

### Ethics statement

The treponemal DAL-1 strain was propagated in rabbits at the Veterinary Research Institute in Brno, Czech Republic. The handling of animals in the study was performed in accordance with the current Czech legislation (Animal Protection and Welfare Act No. 246/1992 Coll. of the Government of the Czech Republic). These specific experiments were approved by the Ethics Committee of the Veterinary Research Institute (Permit Number 20–2014).

### *T*. *pallidum* protein sample preparation

Cell lysis of the purified *T*. *pallidum* extract was performed by conducting three consecutive freeze-thaw cycles, followed by ultrasonication on ice with an amplitude of 50% and a pulser frequency of 2 seconds for 2 minutes (Sonics, Vibra cell; Newton USA). Protein concentration was determined by loading a small fraction of the lysed sample on a high performance liquid chromatographic (HPLC) reversed phase C4 system that was calibrated using a serial dilution of a protein standard mixture. Protein concentrations were determined based on the area under the curve (AUC at 214 nm). Approximately 400–500 μg of protein was extracted from each biological replicate; a large proportion of this amount was host protein in the form of albumin. Samples were acetone precipitated by adding 6 volumes of LC-MS grade acetone (Biosolve, Valkenswaard, Netherlands) and incubated overnight at -20°C. In all cases, lo-bind Eppendorf tubes (Eppendorf, Hamburg, Germany) were used to ensure high recovery rates of proteins/peptides.

### Protein enzymatic digestion

Following protein precipitation, protein samples were re-suspended in 50 mM Tris-HCl/6 M urea/5 mM DTT/10% beta-mercaptoethanol (25 μL/100 μg protein) at pH 8.7. For the denaturation and reduction process all samples were incubated at 65°C during 1 hour. Subsequently, proteins in all fractions were diluted in 50 mM Tris-HCl/ 1 mM CaCl_2_ (75 μL/100 μg protein) and alkylated by adding 200 mM iodoacetamide (10 μL/100 μg protein) during 1 hour at room temperature. Proteomics-grade modified trypsin (Promega, Madison, Wisconsin, United States) was added at a 30:1 protein-to-enzyme ratio. After incubation at 37°C for 18 hour the digestion was stopped by freezing the samples.

### Peptide separation by reversed phase C18 at high pH (1^st^ dimension)

After tryptic digestion, peptides were separated in a first dimension based on hydrophobicity at high pH by using a reversed phase C18 column (X!Select, CSH, RP-C18, 2.1 x 150 mm, 3.5 μm, Waters) connected to a Waters Alliance e2695 HPLC bio-system and a Waters 996 PDA detector (Waters Corporation, Milford, MA, USA). Solvent A contains 200 mM ammonium formate at pH 10, while solvent C contains 100% water and solvent D 100% acetonitrile (ACN) (LC-MS grade, Biosolve, Valkenswaard, Netherlands). During the chromatographic run, an ACN gradient was performed, while continuously 10% of solvent A was added to become an overall pH of 10 during the entire run. The following gradient was used at a constant flow rate of 200 μL/min: 5% to 15% D over the first 5 min, 15% to 40% D over 80 min, 40% to 90% D over 8 min, 5 min 90% D, and 90% to 5% D over 2 min. In total, 30 fractions were collected starting from 10 to 100 min with an interval of 3 min/fraction. The peptide concentration of the different fractions was determined based on the area under the curve (AUC at 214 nm). Fractions were pooled in a concatenated way (e.g. fractions 1, 11 and 21) to obtain optimal orthogonality, yielding in total 10 fractions for further analysis. Collected fractions were lyophilized and re-suspended in RP mobile phase (97% water, 3% ACN, 0.1% FA).

### Peptide separation by micro-capillary reversed phase C18 (2^nd^ dimension)

Peptide fractions were separated in a second dimension using an Agilent 1100 series micro-capillary HPLC system (Agilent Technologies, Waldbronn, Germany). For each fraction 15 μg of peptides was injected on a Zorbax 300SB-C18 guard column (0.3 mm x 5 mm; particle size 3.5 μm; Agilent Technologies) serially connected with a Zorbax 300SB-C18 analytical RP column (0.3 mm x 150 mm; particle size 3.5 μm; Agilent Technologies). Samples were online desalted by loading the peptides on the guard column before the ACN gradient was started. Solvent A contained 0.1% formic acid (FA) in water while solvent B contained 0.1% FA in 90% ACN /10% water. Following ACN gradient was performed using the capillary pump with a constant flow rate at 6 μL/min: 5% to 60% B in 56.7 min, ramp to 90% B over 3.3 min persistent 90% B for 5 min, 85% B for 5 min and back to equilibrating conditions of 3% B. Starting from minute 5 until minute 51.7 of the chromatographic run, 350 spots (800 nl/spot) for each fraction were spotted on an Opti-TOF MALDI-target (28 columns x 25 rows; 8 sec interval; 700 spots; 2 runs per target) (Applied Biosystems, Inc.). Afterwards, each spot was covered with matrix (2 mg/ml α-cyano-4-hydroxycinnamic acid in 70% ACN; internal calibrant: 93 pmol/ml human [Glu^1^]-fibrinopeptide B) using an external syringe pump with a 4 second interval (800 nl matrix/spot) at a flow rate of 12 μL/min.

### MALDI-TOF/TOF MS/MS analysis

Spotted fractions were offline analysed using a MALDI ABi4800 proteomics analyser (Applied Biosystems). MALDI-TOF MS-analysis (reflectron mode; laser intensity: 3400; 25 x 20 laser shots per spot; mass-range 800–3000 Da) was performed first, after which precursors were selected with a signal-to-noise (S/N) ratio above or equal to 100. [Glu^1^]-fibrinopeptide B (m/z 1570.667) was used as internal standard to calibrate MS-spectra. MALDI-TOF/TOF MS/MS-analysis was performed on the selected MS precursors. A maximum of 50 unique precursors per spot were selected for fragmentation, starting from the precursors with the lowest S/N- ratio. These precursors were ionized (laser intensity: 4300; 25 x 20 laser shots per spot) and fragmented in a collision cell (CID, 1 kV collision energy).

### MALDI-TOF/TOF MS/MS spectral data analysis

Spectra from each sample were extracted by Peak Explorer and screened against a *T*. *pallidum* database UniProt proteomes IDs UP000014259 and UP000000811 using the MASCOT search engine (Matrix Science; version 2.1.03) based on the digestion enzyme trypsin. We chose to screen against the Nichols strain database since the DAL-1 strain proteome is not well annotated and the genetic differences between the strains are minimal and described [50]. The latter database is generally used as the treponemal reference database while the former is a more recent version. Carbamidomethylation of cysteines was listed as fixed modification, while oxidation of methionine was set as a variable modification. A maximum of two missed cleavages of trypsin was tolerated. Mass tolerance was set to 200 ppm for the precursors and 0.20 Da for the fragment ions. The MudPIT scoring algorithm of MASCOT was used. Scaffold Q+ (version Scaffold 4.0.5, Proteome Software Inc., Portland, OR) was used to validate MS/MS-based peptide and protein identifications. Because the *T*. *pallidum* proteome contains several small proteins with just one or a few detectable tryptic peptides, protein identifications based on one unique peptide were only allowed if they fulfilled certain stringent conditions; these criteria were comprised by the peptide prophet algorithm that was performed by Scaffold Q+. Protein identifications were accepted if they could be established at greater than 95.0% probability according to the protein prophet algorithm.

Protein abundances were estimated based on the spectral counts (SC) of each identified protein by calculating the normalized spectral abundance factor (NSAF) as previously described [51]. In short, this approach includes a normalisation step based on (1) the observable peptides (OP) and (2) on the total number of identified peptides. The NSAF values reflecting an average of the biological and technical runs of each detected proteins are provided in S3 Table. Pearson’s correlation test and Mann Whitney test were calculated to compare the cDNA/DNA signal data to the NSAF protein abundance data. A P-value of < 0.05 was considered statistically significant. All analyses were performed in Stata 12 (StataCorp LP, College Station, TX, USA).

In order to determine whether the identification methodology was stringent enough, the false discovery rate (FDR) was defined on protein level by using a concatenated database consisting of the target spectral database and a shuffled database. Calculation of FDR was performed as follows: 2x false positive identifications / (false positive identifications + true positive identifications) [52]. For all samples, the FDR on protein level had to be less than 5%. Spectra were also screened against the mammalian Swissprot database containing human (*Homo sapiens*) and rabbit (*Oryctolagus cuniculus*) proteomes for spectra verification to prevent assignment of peptides with a conserved amino acid sequence.

### Orbitrap Velos LTQ MS/MS analysis

#### Nano reverse phase liquid chromatography and mass spectrometry

The peptide mixtures were separated in the second dimension by reverse phase chromatography on an Eksigent nano-UPLC system using an Acclaim C18 PepMap100 nano-Trap column (200 μm x 20 mm, 5 μm particle size) connected to an Acclaim C18 analytical column (75 μm x 150 mm, 3 μm particle size) (Thermo Scientific, San Jose, CA). Peptide fractions were dissolved in mobile phase A, containing 2% ACN and 0,1% formic acid and spiked with 20 fmol [Glu^1^]-fibrinopeptide B. A linear gradient of mobile phase B (0,1% FA in 98% ACN) in mobile phase A (0,1% FA in 2% ACN) from 2 to 45% B in 35 min followed by a steep increase to 95% mobile phase B in 2 min was used at a flow rate of 350 nl/min. The nano-LC was coupled online with the mass spectrometer using a PicoTip Emitter (New objective, Woburn, MA) coupled to a nanospray ion source.

The LTQ Orbitrap Velos (Thermo Scientific, San Jose, CA) was set up in a data dependent MS/MS mode where a full scan spectrum (350–5000 m/z, resolution 60000) was followed by a maximum of ten CID tandem mass spectra (100 to 2000 m/z). Peptide ions were selected as the twenty most intense peaks of the MS scan. Collision induced dissociation (CID) scans were acquired in the LTQ ion trap part of the mass spectrometer. The normalized collision energy used was 35% in CID. A dynamic exclusion list of 45 sec for data dependent acquisition was applied.

### Orbitrap Velos LTQ MS/MS spectral data analysis

Spectra from each sample were extracted by Proteome discoverer software (Thermo Scientific, San Jose, CA) and screened against a *T*. *pallidum* database (UniProt ID proteomes IDs UP000014259 and UP000000811) using the MASCOT search engine (Matrix Science; version 2.1.03) based on the digestion enzyme trypsin. Carbamidomethylation of cysteines was listed as fixed modification, while methionine oxidation was set as variable modification. A maximum of two missed cleavages of trypsin was tolerated. Mass tolerance was set to 10 ppm for the precursors and 0.8 Da for the fragment ions. The MudPIT scoring algorithm of MASCOT was used. Further protein identification, quantification and validation procedures were conducted as mentioned above for the MALDI-TOF/TOF analysis. All Orbitrap LTQ mass spectrometric data are available at PeptideAtlas [53]. The identifier is PASS00903.

### Identification of known and predicted *T*. *pallidum* membrane proteins

Initially, all potential membrane proteins were identified from the 557 *T*. *pallidum* proteins detected by mass spectrometry by: (1) analyzing annotated functions (and subcellular localizations, if available) from all published *T*. *pallidum* ssp. *pallidum* genome sequences (http://www.ncbi.nlm.nih.gov/genome/?term=treponema+pallidum%5Borgn%5D), (2) identification of lipoproteins based on previous bioinformatic analyses performed by Setubal *et al*. [26], (3) identification of rare outer membrane proteins based on previous bioinformatic analyses performed by Cox *et al*. [27], and (4) by additional review of experimental findings in the scientific literature. Next, all potential membrane proteins (and proteins annotated as ‘uncharacterized’, ‘hypothetical’ or ‘conserved hypothetical’) were analyzed using 5 bioinformatic prediction tools. The SignalP 4.1 server (http://www.cbs.dtu.dk/services/SignalP/) [54] and the LipoP 1.0 server (http://www.cbs.dtu.dk/services/LipoP/) [55] were used to predict the presence and location of potential signal peptide cleavage sites and lipoprotein signal peptides, respectively. PSORTb version 3.0.2 (http://www.psort.org/psortb/) [56] was used to predict protein subcellular localization. TMHMM server version 2.0 (http://www.cbs.dtu.dk/services/TMHMM-2.0/) [57] and PRED-TMBB (http://bioinformatics.biol.uoa.gr/PRED-TMBB/) [58] were used for predicting the presence and location of transmembrane alpha-helices and beta strands, respectively. Proteins with unclear subcellular localization predictions using the above bioinformatic pipeline were further analyzed using the following eight prediction tools. CELLO version 2.5 (http://cello.life.nctu.edu.tw/) [59] was used to predict subcellular localization. Philius (http://www.yeastrc.org/philius/pages/philius/runPhilius.jsp) [60], Phobius (http://phobius.sbc.su.se/) [61], Octopus/Spoctopus (http://octopus.cbr.su.se/index.php) [62], HMMTOP version 2.0 (http://www.enzim.hu/hmmtop/html/submit.html) [63], and TMpred (http://www.ch.embnet.org/software/TMPRED_form.html) were used for the prediction of transmembrane alpha-helices BOMP (http://services.cbu.uib.no/tools/bomp) [64] and TMBETADISC-RBF (http://rbf.bioinfo.tw/~sachen/OMPpredict/TMBETADISC-RBF.php) [65] were used for the prediction of beta-barrel outer membrane proteins.

### Assignment of orthologous functional categories and cellular localization

The eggNOG version 4 database (retrieved 21/04/15) was used to assign COG and NOG categories to all genomes. First all proteins per sample were compared to the eggNOG database using USEARCH version 7.0.959 with an e-value of 1e-30 and a bit-score cut-off of 70% of the top hit to ensure only close matches were retrieved and reduce the likelihood of spurious annotations. An eggNOG membership is assigned to each protein if 70% of the UBLAST hits belong to the same eggNOG member. Distinctions are then made between proteins with no UBLAST hit to any eggNOG sequence (no_hit) and over 70% of hits to a member that is not assigned an eggNOG code (none). Annotations are also clustered at the 25 higher COG functional category levels as per the eggNOG assignments. Classification of proteins according to their cellular location was achieved using data extraction from online databases (Swissprot) and the methods as outlined for the membrane localized proteins.

## Results and Discussion

### Mass spectrometry analysis

In short, from the three biological replicates, a total of 6033 *T*. *pallidum* peptides were detected corresponding to 557 proteins and 54% of the total predicted proteome (S1 Table). Proteins ranged in size from 6–173 kDa with a pI range of 4.15 to 12.05. Acquired spectra were screened against two Nichols strain UniProt proteomes whereby three extra proteins (TP0248, TP0651 and TP0922) were uncovered compared to when solely screened against the Nichols reference UniProt proteome (ID: UP000000811) [13]. In the resequenced proteome (ID: UP000014259) [14] three of these proteins were below the 150bp annotation limit. We found 57/102 proteins containing previously reported sequencing errors [14] compared to the original genome analysis [13], including two genes with an authentic frameshift, 14 reannotated gene fusions and 5 novel ORFs reannotated in the new proteome (S2 table).

**Fig 1:**
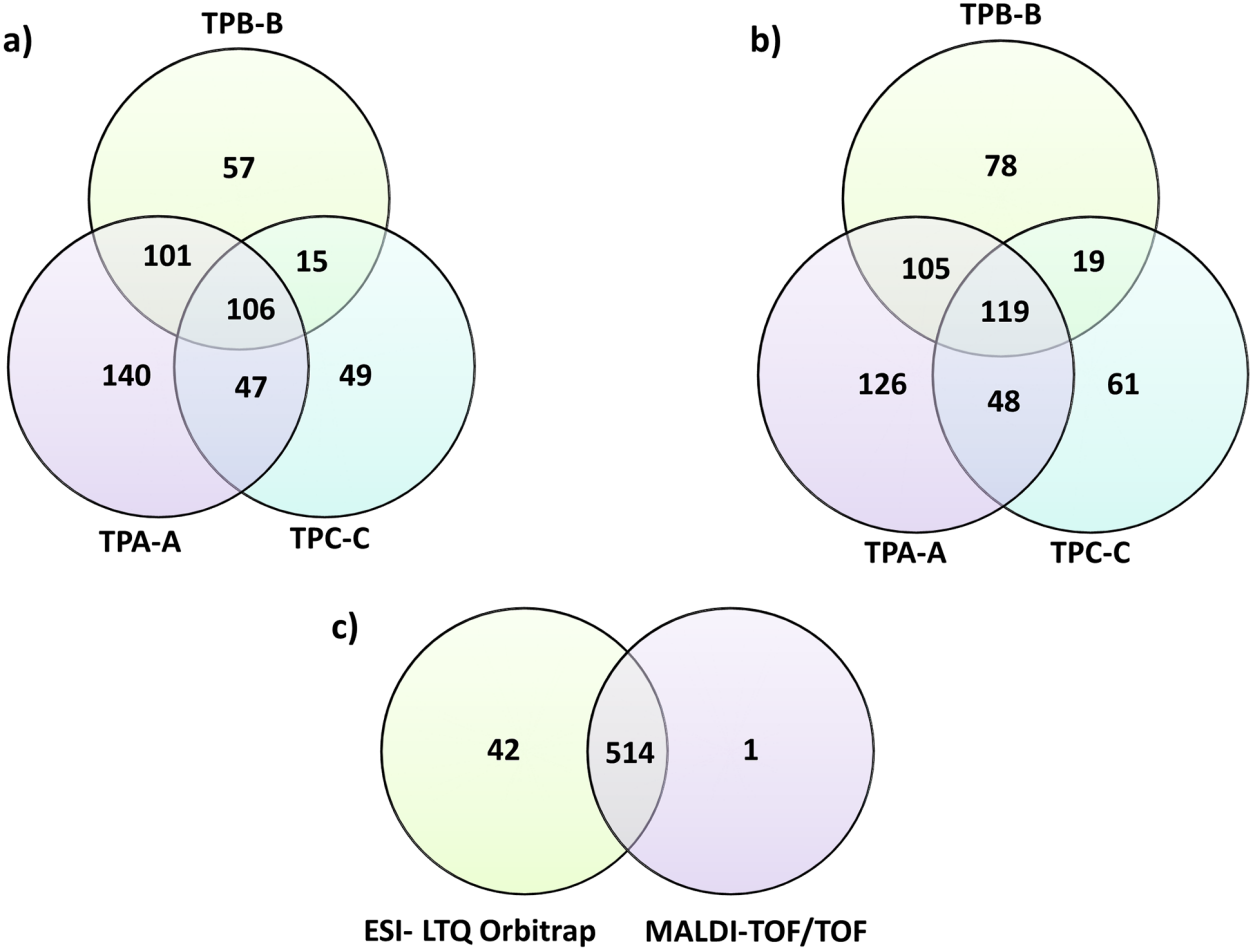
Venn Diagrams depicting the total number of unique *T*. *pallidum* proteins identified per rabbit biological replicate (N = 3) analyzed by (A) Matrix-assisted laser desorption/ionization time of flight and (B) Electrospray Ionization LTQ- Orbitrap Velos MS/MS. All spectra were screened against the UniProt databases (ID: UP000000811 & UP000014259), with a peptide and protein identification confidence interval of 95%. There was considerable overlap between the complementary MS analytical methods whereby an additional 42 treponemal proteins were found in the Orbitrap analysis as depicted in diagram (C).

**Fig 2:**
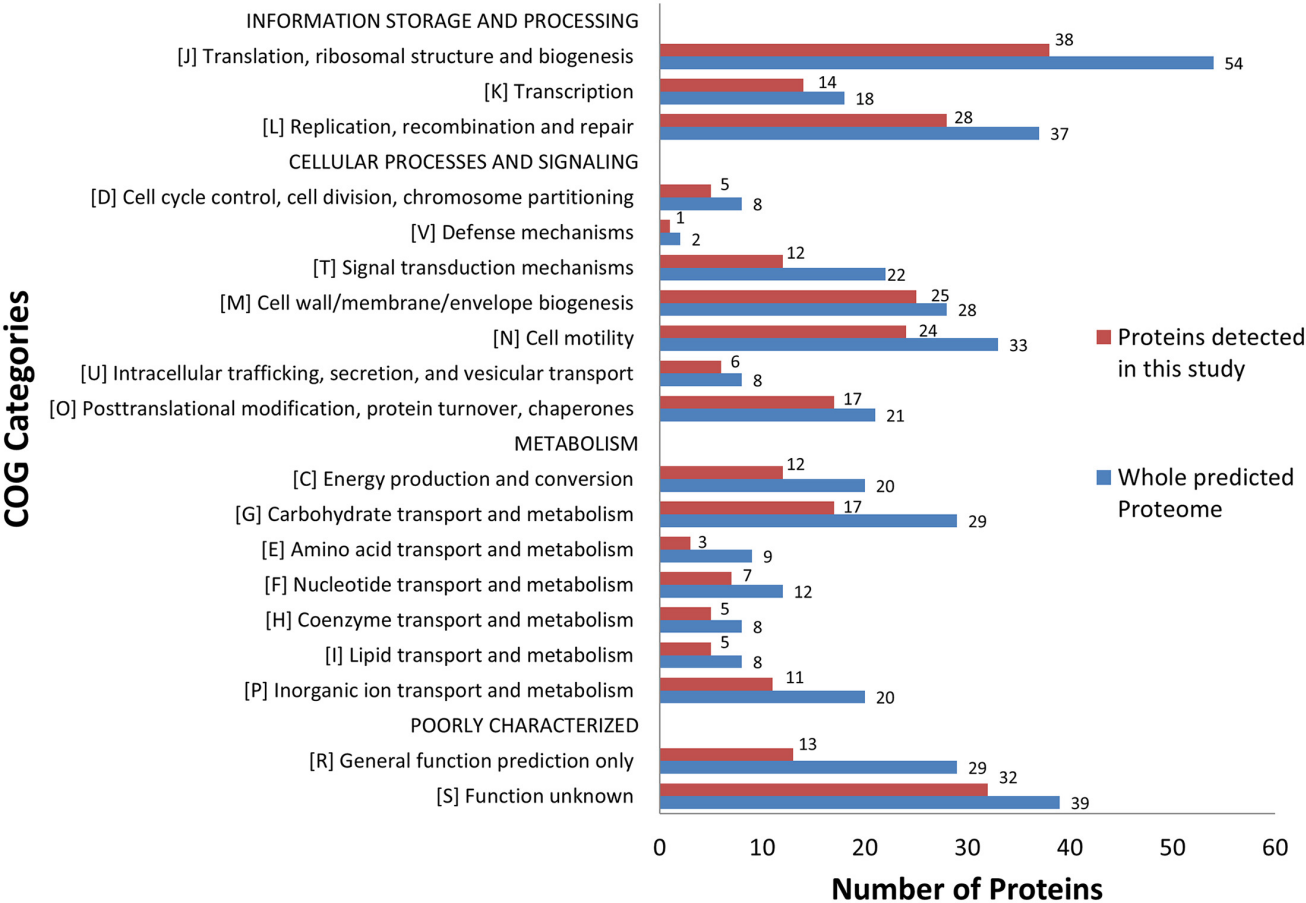
Bar Diagram depicting the distribution of the detected and undetected *T*. *pallidum* proteins distributed in 19 COG functional categories. Blue bars represent MS detected proteins in this study. Red bars represent all predicted proteins in the *T*. *pallidum* proteome. The functional category was automatically determined for genes that could be placed in Clusters of Orthologous Groups (COGs). For genes with more than one COG category both categories were used.

Pertaining to the individual samples, 394/398 (TPA-A), 279/321 (TPB-B) and 217/247 (TPC-C) proteins were uniquely identified by MALDI-TOF/TOF and ESI- MS/MS analysis, respectively, of which 106 (MALDI-TOF/TOF) and 119 (ESI- MS/MS) proteins were present in all three biological samples (Fig 1A/1B). Only 31 proteins were found with less than 2 peptide identifications in one biological and one technical run (S3 Table). For the individual MS analyses (MALDI- TOF/TOF *versus* ESI- LTQ Orbitrap MS/MS detection), 514 proteins were detected by both methods (Fig 2C). Only one and 42 additional proteins were exclusively identified by MALDI- TOF/TOF MS/MS analysis and ESI-MS/MS analysis, respectively (Fig 2C) indicating that we are possibly approaching the upper limit of the detectable *T*. *pallidum* proteome and that the non-detected proteins are 1) not expressed, or 2) are expressed at a very low level. All *T*. *pallidum* designated spectra were rescreened against human and rabbit UniProt protein databases and no overlap was found.

A previous proteomics study of *in vivo* rabbit expressed *T*. *pallidum* Nichols strain bacteria [16] detected 88 proteins using MALDI-TOF MS with peptide mass fingerprinting. We detected 58 of these proteins, therefore, to date 58% of the whole *T*. *pallidum* predicted proteome has been detected using MS methods. We failed to detect 30 of these previously identified proteins as outlined in Table 1. The protein detection differences between the studies could be attributed to different experimental methods, for example gel-based *versus* liquid chromatographic separation, which may favor the detection of proteins with certain physiochemical characteristics. Although the differences on the genomic level between the two strains are minimal [50], different duplication rates or other strain characteristics could contribute to different protein expression profiles found between these studies.

**Table 1:** *T*. *pallidum* proteins found in previous proteome investigation (McGill *et al*.) [16] but not detected in this study.

TP number	UniProt Accession Number^#^	Protein Name(s)	Molecular Weight (kDa)	COG category code	cDNA/DNA signal ratio Smajs *et al*. [15]
TP0112	O83150	Aminopeptidase C (PepC)/Bleomycin hydrolase	51	none	1.0
TP0139	O83175	Trk family potassium (K+) transporter, NAD+ binding protein	25	P	0.6
TP0201	R9UV93	50S ribosomal protein L5	21	none	1.8
TP0216	R9UVA6	Chaperone protein DnaK/Heat shock 70 kDa protein	68	none	4.5
TP0239	R9UU25	50S ribosomal protein L10	20	none	1.9
TP0259	O83283	Peptidoglycan binding (LysM domain-bacterial cell wall degradation)	29	S	5.4
TP0298	R9UW99	ABC superfamily ATP binding cassette transporter, membrane protein/ Membrane lipoprotein TpN38	38	none	3.3
TP0349	R9UUA6	Peptidyl-prolyl cis-trans isomerase/ Metallochaperone SlyD	18	O	2.2
TP0356	O83375	Putative RNA-binding protein	12	none	10.7
TP0424	R9UWN5	Two-sector ATPase, V(1) subunit E	19	none	4.7
TP0435	R9UWP1	Copper resistance lipoprotein NlpE/17 kDa lipoprotein	17	none	5.1
TP0448	R9UUM3	Uracil phosphoribosyltransferase	41	F	0.3
TP0453	O67998	Outer membrane protein/30kLp	32	none	1.0
TP0476	R9UWS9	Acetate kinase/Acetokinase	49	none	1.3
TP0505	O83518	Hexokinase	48	none	1.8
TP0537	R9UTC2	Triosephosphate isomerase	27	G	1.9
TP0554	O83565	Phosphoglycolate phosphatase	25	R	1.1
TP0556	R9UWZ4	Aspartate—ammonia ligase/Asparagine synthetase A	37	E	1.8
TP0584	R9UTG3	Uncharacterized protein	54	none	0.8
TP0611	O83620	ABC superfamily ATP binding cassette transporter, ABC protein	28	none	1.2
TP0655	O83661	Spermidine/putrescine ABC superfamily ATP binding cassette transporter, binding protein	40	E	0.6
TP0734	R9UXE0	Purine-nucleoside phosphorylase	25	F	1.6
TP0769	R9UTV1	Treponemal membrane protein B/ Antigen TmpB	37	S	5.2
TP0821	R9UVI4	Lipoprotein/ Membrane lipoprotein TpN32; 29 kDa protein	29	P	1.5
TP0823	O83795	Desulfoferrodoxin (Rbo)	14	C	3.2
TP0862	O83834	Peptidyl-prolyl cis-trans isomerase	28	none	5.3
TP0925	R9UUA1	Nitrogenase (Flavodoxin)	16	C	9.4
TP0964	O83930	ABC transporter, ATP-binding protein	25	V	0.9
TP0971	R9UX80	Tp34 lipoprotein/ 34kDa membrane antigen	22	none	12.6
TP1013	R9UUH7	10 kDa chaperonin/GroES protein/Protein Cpn10	9	O	4.6

Legend:

^#^: UniProt Proteome ID: UP000014259; Clusters of Orthologous Genes (COG)

### Detection of possible *T*. *pallidum* heterogeneous sites at the protein level

All *T*. *pallidum* protein sequences were screened for possible heterogeneous sites by searching the spectral databases for amino acid sequences containing sites designated with ‘X’, meaning ‘undetermined amino acid site’. Heterogeneous sites were defined as differing amino acids located at the same coordinate ‘X’ in the same protein sequence. A total of 25 *T*. *pallidum* proteins contained sites designated as ‘X’, of which four proteins were identified with heterogeneous peptide matches at site ‘X’ (Table 2). Amino acid sequence diversity was found within one sample for three proteins, TP0082 (TPC-C), TP0248 (TPC-C) and TP0922 (TPB-B). Protein TP0692 contained two peptides with heterogeneous sites within two samples (TPA-A/TPC-C). This is the first account of sequence heterogeneity at the protein level for these particular proteins. Although the amino acid sequence designation is of high confidence (95%), cautious interpretation of these results is warranted as *de novo* peptide sequencing was not utilized so these analyses could represent falsely identified sites, therefore, further research is advised. *Treponema pallidum* intra-strain nucleotide sequence heterogeneity has been reported previously [14,66,67], including *tprK* [22,31,32,66,68,69] and heterogeneity in four DAL-1 strain genes related to chemotaxis and metabolism [66]. The functional relevance of this observed intra-strain variability in these proteins in currently unknown.

**Table 2:** Potential heterogeneous sites identified within peptides of MS detected *T*. *pallidum* proteins.

					Amino acid detected at site 'X' in peptide sequence					
					MALDI TOF/TOF			ESI-LTQ-Orbitrap		
					Sample			Sample		
UniProt Accession Number^#^	TP number	Protein Name	Molecular Weight (kDa)	Identified Peptide Sequence	TPA-A	TPB-B	TPC-C	TPA-A	TPB-B	TPC-C
O88098	TP0082	Formate hydrogenlyase transcriptional activator (FhlA)	66	(R)LYPIXNAR(K)			T			D
O83276	TP0248	Uncharacterized lipoprotein	15	(R)AYELXER(S)			T			H
O83690	TP0692	Protein RecA	44	(K)TLRRXASR(G)	Y		H	T		P
O83892	TP0922	Uncharacterized protein	33	(R)ALXGNDPSAAVR(V)		V			A	

Legend:

^#^: UniProt Proteome ID: UP000014259; Matrix-assisted laser desorption ionization-time of flight (MALDI-TOF/TOF); electrospray ionization LTQ-Orbitrap tandem mass spectrometry (ESI-LTQ-Orbitrap), X = undetermined amino acid site

### Bioinformatic characterization of detected *T*. *pallidum* proteins

Bioinformatic analyses assigned 279 detected proteins to 19 higher Clusters of Orthologous Genes (COG) functional category levels according to their eggNOG assignments. Distributional description of these proteins and their categorical frequencies are depicted in Fig 2 and extensive descriptions, including COG/NOG codes for all detected proteins, can be found in S2 Table. Of the proteins that were delegated into a clear functional category, the highest representative categories were ‘J’ (translation, ribosomal structure and biogenesis) (17%) and ‘L’ (replication, recombination and repair) (12%). High category coverage was found for the categories ‘M’ (cell wall/membrane/envelope biogenesis) and ‘O’ (posttranslational modification, protein turnover and chaperones) with 25/28 and 17/21 proteins found, respectively. Forty-five proteins fell under category ‘S’ or ‘R’, indicating poor functional characterization. A total of 9 proteins had no UBLAST hit to any eggNOG sequence (category ‘no_hit’), of which 5 proteins were ribosomal and 4 were uncharacterized. Many proteins (N = 275) were at least 70% homologous to a protein member not assigned an eggNOG code (category ‘none’) indicating that the *T*. *pallidum* proteome is very unique compared to other organisms. Six proteins were categorized under multiple COG categories. In almost all of the COG categories, more than half of the predicted proteins were detected, supporting the theory that *T*. *pallidum* has shed its unnecessary genes during its evolution [13].

The *T*. *pallidum* Nichols and SS14 strain genomes differ minimally [14], thus in the case of genetically congruent ORFs we extrapolated recent *T*. *pallidum* strain SS14 hypothetical protein function re-annotations [28] to 22 ‘uncharacterized/hypothetical’ proteins detected in this analysis. In total, 114 proteins remained classified as ‘uncharacterized proteins/hypothetical proteins’. This category did not include 17 proteins with ambiguous “putative” membrane protein descriptions. A previous study [16] detected eight of these uncharacterized proteins, meaning that this is the first account of 106 ‘uncharacterized/hypothetical’ proteins at the protein level. This uncharacterized area of the *T*. *pallidum* proteome may contain novel proteins with important roles in pathogenesis and even represent novel biomarker, treatment or vaccination targets.

### Predicted cellular localization of detected *T*. *pallidum* proteins

**Fig 3:**
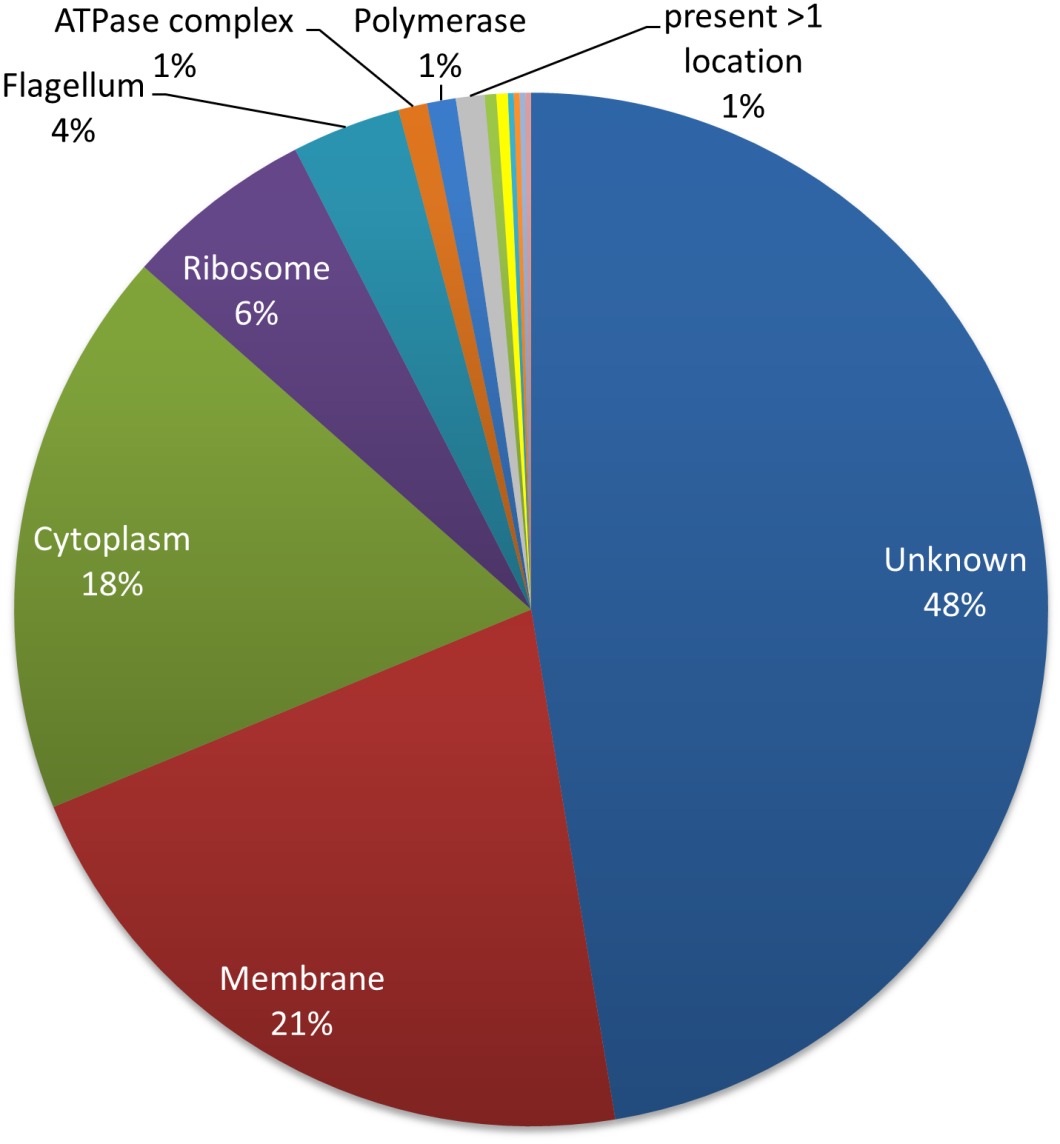
Pie diagram depicting the subcellular localization of *T*. *pallidum* detected by MS analysis (N = 557). Almost half of the detected proteins (N = 265; 48%) did not have an annotated cellular location. Of the known locations, membrane (N = 116; 21%), cytoplasm (n = 99; 18%), ribosomal (N = 33; 6%) and flagella (N = 19; 3%) were the most represented cellular localizations.

The global classification of detected proteins according to their cellular localization was achieved by screening online databases such as UniProt and by reviewing relevant literature. The cellular localization of the proteins was predicted for 292/557 proteins; these were largely localized in the cytoplasm (N = 97, 17%), membrane (N = 116, 21%), ribosome (N = 33, 6%) and flagella (N = 19, 3%). A schematic breakdown of the predicted cellular localizations for all detected proteins can be found in Fig 3, with comprehensive information for each protein provided in S2 Table.

**Fig 4:**
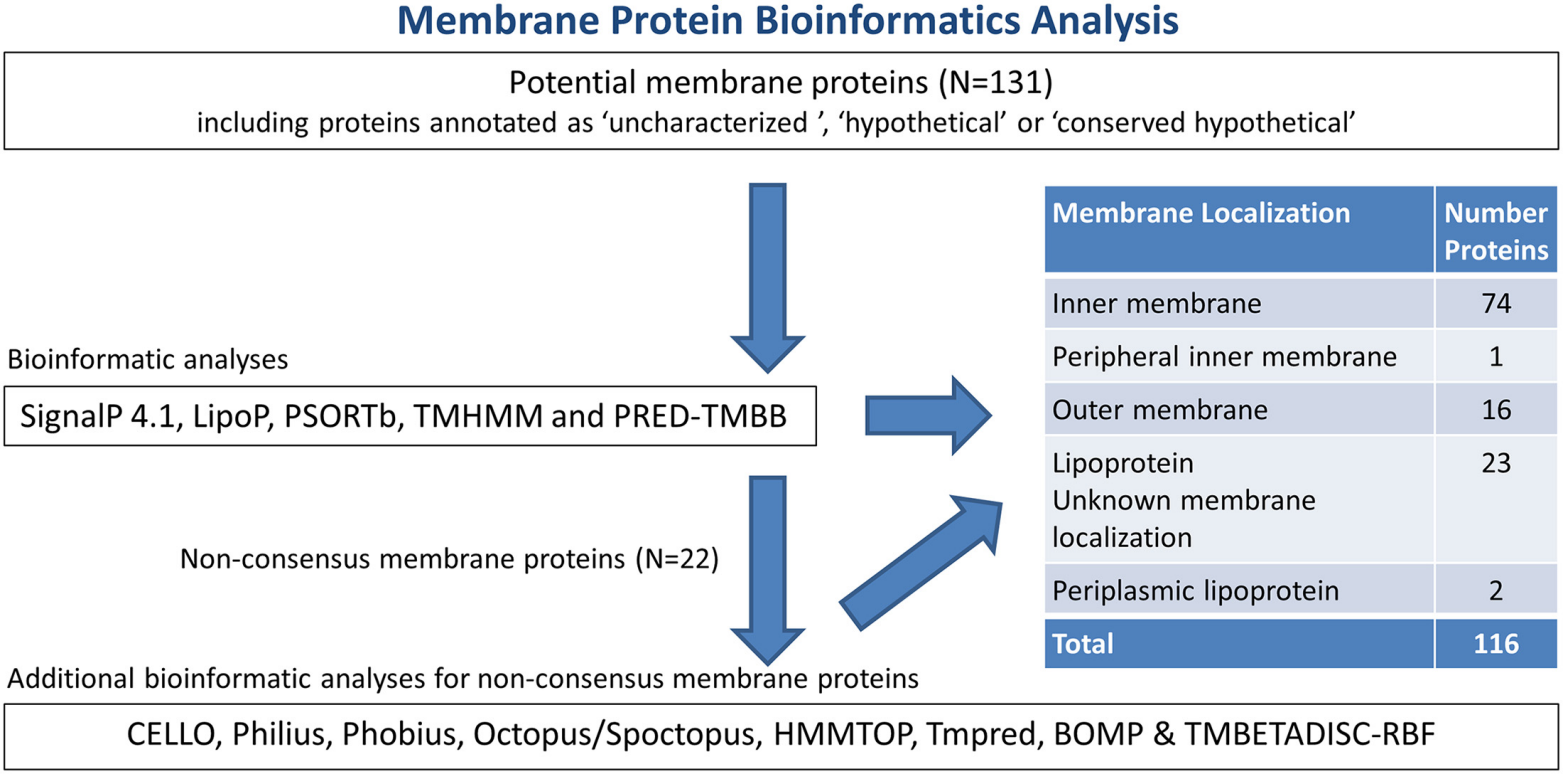
Bioinformatics pipeline analysis for potential *T*. *pallidum* membrane proteins.

Detected proteins were subjected to additional bioinformatic pipeline analyses in order to identify potential membrane proteins as detailed in the methods section. In short, all potential membrane proteins (N = 131; including proteins annotated as ‘hypothetical’) were analyzed using five bioinformatic prediction tools: SignalP 4.1 [54], LipoP [55], PSORTb [56], TMHMM [57] and PRED-TMBB [58]. Proteins with unclear subcellular localization predictions using the above bioinformatics pipeline (N = 25) were further analyzed using an additional eight prediction tools including CELLO [59], Philius [60], Phobius [61], Octopus/Spoctopus [62], HMMTOP [63], Tmpred, BOMP [64] and TMBETADISC-RBF [65]. A results summary of this analysis can be found in Fig 4 and S4 Table.

In total, 116 proteins were designated as ‘membrane’ localized, with a majority (64%; N = 74) located within the inner membrane. Sixteen proteins (14%) were predicted to be located in the outer membrane (OM) (Table 3). The OM localization of five detected proteins has been experimentally investigated: (TP0117 (TprC)/ TP0131 (TprD) [27,37,70], TprI (TP0620) [38], TP0126, an OmpW homologue [71] and TP0326, a BamA homologue [27,72–74].

**Table 3:** Predicted *T*. *pallidum* outer membrane proteins detected by MS analysis.

TP number	UniProt Accession Number^#^	Protein Name	References
TP0011	O83055	Tpr protein B	Cox *et al*. 2010 [27]
TP0117*	O88138	Tpr protein C (TprC)/ Tpr protein D (TprD)	Anand *et al*. 2012 [37]; Cox *et al*. 2010 [27]; Centurion-Lara *et al*. 2013 [70]
TP0126	R9UV28	Uncharacterized protein	Giacani *et al*., 2015 [71]
TP0131*	O88138	Tpr protein C (TprC)/ Tpr protein D (TprD)	Anand *et al*. 2012 [37]; Cox *et al*. 2010 [27]; Centurion-Lara *et al*. 2013 [70]
TP0155	O83190	M23B subfamily peptidase	Cameron *et al*. 2004 [75]; Cox *et al*. 2010 [27]
TP0313	O83335	Tpr protein E (TprE)	Cox *et al*. 2010 [27]
TP0324	R9USU0	Putative outer membrane protein	Cox *et al*. 2010 [27]
TP0326	O83346	Outer membrane protein	Cameron *et al*. 2000 [74]; Cox *et al*. 2010 [27]; Desrosiers *et al*. 2011 [72]
TP0421	O83436	Uncharacterized protein	Cox *et al*. 2010 [27]
TP0620	R9UV07	Tpr protein I (TprI)	Giacani *et al*., 2005 [76]; Centurion-Lara *et al*. 2013 [70]; Cox *et al*. 2010 [27]; Anand *et al*. 2015 [38]
TP0621	F7IWA5	Tpr protein J (TprJ)	Cox *et al*. 2010 [27]
TP0855	R9UWW7	Putative outer membrane protein	Cox *et al*. 2010 [27]
TP0858	R9UXR8	Uncharacterized protein	Cox *et al*. 2010 [27]
TP0865	R9UU78	Putative outer membrane protein	Cox *et al*. 2010 [27]
TP0923	R9UX30	Putative outer membrane protein	
TP0969	R9UUH1	Putative outer membrane protein	Cox *et al*. 2010 [27]

Legend:

^#^: UniProt Proteome ID: UP000014259,

*tprC and *tprD* alleles are identical at the genomic level in DAL-1 strain [50]

The other 11 predicted OM proteins in this analysis were: TprB (TP0011), M23B subfamily peptidase (TP0155), TprE (TP0313), TprJ (TP0621), TP0421, TP0858, TP0324, TP0855, TP0865, TP0923 and TP0969.

A previous *in silico* prediction analysis of the *T*. *pallidum* genome revealed 46 predicted lipoproteins [26]. Our analysis also detected 25 lipoproteins, 23 with unknown membrane locations and two located within the periplasm (TP0796; TP0171), including the 15 kDa (Tpp15) lipoprotein (TP0171) and 47 kDa membrane antigen (TP0574). In spirochetes, lipoproteins are highly expressed molecules primarily localized in the periplasm anchored to the outer leaflet of the cytoplasmic membrane [9] where they are thought to modulate immune responses from both innate and adaptive immunity [43,77].

There were ambiguities regarding the subcellular localization of 13 proteins after analysis with the additional prediction tools (S4 Table), including TprG (TP0317), TprH (TP0610), ABC superfamily ATP binding cassette transporter (TP0786), flagellar hook length control protein FliK (TP0729) and two TolC-like proteins (TP0967 and TP0968). Of the 49/116 reported membrane proteins that could be designated to a COG category, two categories were most represented: ‘P’ (inorganic ion transport and metabolism) (N = 9) and ‘M’ (cell wall/membrane/envelope biogenesis) (N = 6). This agrees with the predicted biological functional location.

Important to note is the fact that most of the protein localization data is based on computational predictions. These types of predictions have an inherent risk of including false positives and also omitting real OM proteins. Further laboratory work is needed to experimentally confirm the cellular locations of these proteins.

### Relative *T*. *pallidum* protein abundance as determined by spectral counting

**Fig 5:**
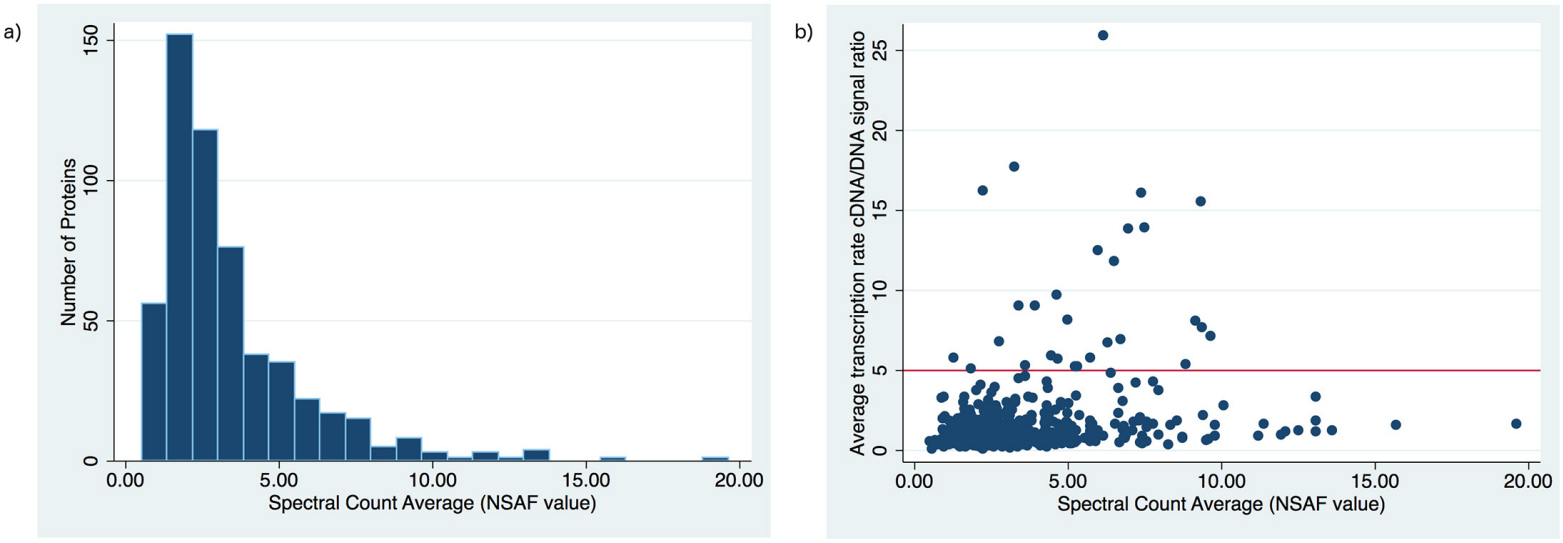
(a) Histogram depicting the distribution of detected *T*. *pallidum* proteins sorted according to their protein abundance amounts (Log expression NSAF values) (b) Scatter Plot of Microarray cDNA/DNA ratio signal *versus* NSAF spectral count average for all MS detected proteins. Statistical analysis revealed no correlation between protein abundance and microarray data (Pearson’s correlation coefficient r = 0.26). Proteins not detected did not have a significantly lower microarray signal as calculated by a two-sample Wilcoxon rank-sum test (P = 0.5). Red line designates cutoff for ‘high abundant’ proteins (NSAF value > 5.0)

We examined the relative abundance of the proteins detected by calculating the NSAF values [78] for the proteins detected in the biological and technical runs; all values are listed in S3 Table and the log distribution of all detected proteins can be found in Fig 5A. This approach is based on the number of observable peptides and normalizes technical variability between samples [78]. A value of ‘1’ represents the mean protein level for all detected proteins. Proteins with an average NSAF value greater than 5.0 were regarded as ‘highly abundant’. A summary of the top 50 highest abundant proteins according to the spectral counting averages is provided in Table 4. High abundant proteins (N = 103) included two proteins related to redox balance, 22 proteins related to translation, two proteins related to chemotaxis and three ABC family transport proteins. Proteins related to motility were found to be high abundant, including flagellar filament proteins (TP0663; TP0792; TP0868; TP0870) and 3 proteins related to flagellar biosynthesis (TP0403; TP0658; TP0718). The fact that proteins related to motility, transport and chemotaxis are highly expressed can be indicative that these processes are essential and highly utilized for cell survival.

**Table 4:** Top 50 most abundant *T*. *pallidum* proteins detected by MS analysis.

TP number	UniProt Accession Number^#^	Protein Name	Molecular Weight (kDa)	Average NSAF value	cDNA/DNA signal ratio Smajs *et al*. [15]	GO Biological Process	Cellular location	COG category
TP0214	R9UVZ6	Uncharacterized protein	7	19.6	1.6		Unknown	no_hit
TP0708	R9UXB8	Uncharacterized protein	18	15.7	1.5		Inner membrane	none
TP0385	O83400	Uncharacterized protein	14	13.6	1.2		Unknown	none
TP0951	R9UVX4	50S ribosomal protein L34	6	13.1	1.8	Translation	Ribosome	no_hit
TP0126a*	R9UTR8	Uncharacterized protein	23	13.1	1.1		Unknown	none
TP0474	R9UUP5	Probable transcriptional regulatory protein	26	13.1	3.3	Regulation of transcription, DNA-templated	Cytoplasm	K
TP0061	R9UTJ4	30S ribosomal protein S18	12	12.5	1.2	Translation	Ribosome	none
TP0048	O83087	Cell shape determination protein CcmA	15	12.1	1.1		Unknown	none
TP0657	R9UWD4	Carbon storage regulator homolog	8	12.0	0.9	mRNA catabolic process; regulation of carbohydrate metabolic process	Unknown	none
TP0409a	R9UT20	Preprotein translocase subunit YajC	14	11.4	1.6		Inner membrane	none
TP0718	R9UXC7	IIISP family Type III (Virulence-related) secretory pathway protein/Flagellar biosynthesis protein FliP	31	11.2	0.8	Bacterial-type flagellum organization; protein secretion	Flagellum/Inner membrane	N
TP0808	R9UWS1	Acyl carrier protein	9	10.1	2.8	Fatty acid biosynthesis; Fatty acid metabolism	Cytoplasm	none
TP0558	O83569	Nickel/cobalt efflux system	32	9.8	0.9		Inner membrane	none
TP0198	R9USD7	30S ribosomal protein S17	10	9.8	1.5	Translation	Ribosome	none
TP0849	R9UU63	50S ribosomal protein L35	8	9.7	7.1	Translation	Ribosome	no_hit
TP0490	O83503	Uncharacterized protein	7	9.6	0.7		Unknown	no_hit
TP0649	O83655	Hemolysin (TlyC)	30	9.5	0.6		Inner membrane	P
TP0213	R9USF3	50S ribosomal protein L17	19	9.4	2.2	Translation	Ribosome	J
TP0072	O83111	Glutaredoxin-related protein	10	9.4	7.6	Cell redox homeostasis	Unknown	none
TP0509	O83522	Alkyl hydroperoxide reductase (AhpC)/Peroxiredoxin	21	9.3	15.5		Unknown	O
TP0919	R9UVS2	Thioredoxin	11	9.2	8.1	Cell redox homeostasis; glycerol ether metabolic process	Unknown	none
TP0362	R9USW9	50S ribosomal protein L28	8	8.9	5.4	Translation	Ribosome	none
TP0368	R9UVP4	Uncharacterized protein	12	8.7	0.8		Inner membrane	none
TP0119	O83156	Amino acid ABC transporter, permease protein (YaeE)	23	8.7	0.7	Transport	Inner membrane	none
TP0195	R9USH3	30S ribosomal protein S3	28	8.5	1.9	Translation	Ribosome	J
TP0604	R9UUZ6	Ribosome-recycling factor	21	8.4	1.6	Protein biosynthesis; translation termination	Cytoplasm	J
TP0045	R9UUU2	Adenosine deaminase	35	8.3	0.4	Hydrolase activity; metal ion binding; deaminase activity; nucleotide metabolic process	Unknown	F
TP0700	R9UV74	Uncharacterized protein	14	8.0	3.7		Unknown	none
TP0315	R9USP4	Uncharacterized protein	23	8.0	0.9		Unknown	none
TP0922	O83892*	Uncharacterized protein	33	7.8	1.6		Unknown	none
TP0887	R9UVP5	30S ribosomal protein S15	10	7.8	4.3	Translation	Ribosome	none
TP0707	R9UTN7	Putative membrane protein	17	7.6	0.6		Inner membrane	none
TP0202	R9UTZ5	30S ribosomal protein S14 type Z	7	7.6	1.8	Translation	Ribosome	no_hit
TP0433	R9UT05	Acidic repeat protein	66	7.6	1.4		Unknown	none
TP0663	R9UV45	Putative flagellar filament outer layer protein FlaA; Tromp-2	27	7.5	13.9	Bacterial-type flagellum-dependent cell motility	Flagellum	none
TP0076	R9UUX9	Sugar ABC superfamily ATP binding cassette transporter, membrane protein	30	7.4	0.4	Transport	Inner membrane	G
TP1029	R9UY97	Uncharacterized protein	25	7.4	0.9		Unknown	none
TP0249	R9UVD9	Flagellar filament outer layer protein (FlaA1)	39	7.4	16.1		Flagellum	N
TP1012	O83976	RNA polymerase sigma factor	35	7.4	0.5	DNA-templated transcription; initiation	Polymerase	K
TP0206a	R9UU00	50S ribosomal protein L30	7	7.4	2.0	Translation	Ribosome	no_hit
TP0194	R9UVW4	50S ribosomal protein L22	14	7.3	1.8	Translation	Ribosome	J
TP0234	R9UU20	50S ribosomal protein L33	7	7.2	4.2	Translation	Ribosome	no_hit
TP0007	R9URZ2	Uncharacterized protein	35	7.1	1.8		Unknown	none
TP0412	O83427	Uncharacterized protein	12	7.1	1.2		Unknown	S
TP0792	R9UU06	Flagellin/ Flagellar filament 33 kDa core protein; Class B	31	7.0	13.8	Bacterial-type flagellum-dependent cell motility	Flagellum	N
TP0654	O83660	Spermidine/putrescine ABC superfamily ATP binding cassette transporter, membrane protein	30	6.9	1.0	Transport	Inner membrane	none
TP0563	O83574	Uncharacterized protein	16	6.9	0.8		Unknown	O
TP0248	O83276*	Uncharacterized lipoprotein TP_0248	15	6.8	1.5		Lipoprotein- unknown membrane localization	S
TP0403	O83418	Flagellar biosynthesis protein FliJ	18	6.8	0.8	Bacterial-type flagellum-dependent cell motility; chemotaxis	Flagellum	none
TP0842	R9UU24	Methionine aminopeptidase	27	6.8	0.8	Protein initiator methionine removal	Unknown	J

Legend:

^#^: UniProt Proteome ID: UP000014259; NA: not available; NSAF normalized spectral abundance factor; no_hit: proteins with no UBLAST hit to any eggNOG sequence; none: over 70% of hits to a member that is not assigned an eggNOG code.

*: the C-terminal 49 amino acids of TP0126a correspond to the N-terminal 49 amino acids of TP0126 due to a genome insertion [29]

In terms of the cellular localizations of high abundant proteins, 18 were membrane localized. Four of these proteins were predicted lipoproteins (TP0248, TP0768, TP0895, TP0789) and two were predicted OM uncharacterized proteins (TP0858, TP0126). Surprisingly, approximately a third of the high-abundant proteins (N = 37) were classified as uncharacterized/hypothetical and seven proteins did not have any significant (70%) match with any other EggNOG sequences indicating these are highly specialized *T*. *pallidum* proteins that may play an important role in unique survival and virulence tactics. The most highly represented COG category of the highly expressed proteins was category ‘J’ (translation, ribosomal structure & biogenesis). A low correlation was found between previous gel-based studies [16,17] that determined protein abundance based on silver staining and protein abundance as determined in this study. For example, some highly abundant gel-detected proteins were not detected in our analysis, such as the uncharacterized protein TP0259 and the Tp34 lipoprotein (TP0971) [16,17]. We found a low correlation between the average transcriptional rate (cDNA/DNA signal) from a previous transcriptome study [15] and the average NSAF value for each detected protein found in this study (Pearson’s correlation coefficient, r = 0.26; P = 0.000). The distribution of these data is depicted in Fig 5B. In general, flagellar proteins and proteins related to flagellar biosynthesis such as flagellar filament outer layer protein (TP0249), putative flagellar filament outer layer protein FlaA (TP0663), and flagellar biosynthetic protein FliP (TP0718) were highly expressed in both studies. There were some notable discordances between the data, such as the high gene expression level measured for lipoprotein antigen Tp47 (TP0574), galactose ABC superfamily ATP binding cassette transporter Tpp38 (TP0684) and the 60kDa chaperonin (TP0030), all of which were found in low abundance at the protein level in this study. Moreover, 27 proteins with high gene expression (cDNA/DNA signal ratios greater than 4.0) were not found in this analysis (Table 1). We theorized that the proteins we failed to detect in our analysis would have a lower mean transcription rate. There was however, no significant cDNA/DNA signal data difference between the detected and undetected proteins as determined by a Two-sample Wilcoxon rank-sum (Mann-Whitney) test (P = 0.5). Other studies have demonstrated low correlations between transcriptome and protein abundance data, as reviewed by Maier *et al*. [79]. Intermediary factors such as translation efficiency and protein half-life play a prominent role in accentuating the lack of a linear association between gene expression and protein abundance.

### *T*. *pallidum* proteins confirmed or predicted to be related to virulence

Thirty-nine proteins implicated in *T*. *pallidum* virulence [20] were detected, including eight members of the *tpr* gene family and a protein related to a beta-barrel assembly machinery (BAM) complex. Brief descriptions of these proteins are detailed below.

### Tpr proteins

Regarding the *tpr* gene family implicated in host-immune evasion [30], 8/12 of these proteins were detected in this analysis, including proteins TprB (TP0011), TprC/D (TP0117/TP0131), TprE (TP0313), TprG (TP0317), TprH (TP0610), TprI (TP0620) and TprJ (TP0621). Proteins TprA (TP0009), TprK (TP0897) and TprL (TP1031) were not detected. There was no unique TprF peptide sequence found in this analysis, although three peptides were uncovered that are homologous for TprC/D, F and I (Table 5). The ORF origin of these peptides cannot be determined. The *tprC* and *tprD* loci contain two identical coding sequences in the reference Nichols and DAL-1 strain genome [13,70], therefore we included the detection of both TprC and TprD since no distinction could be made between the coding ORF origin of these proteins. Even though *tprK* was previously shown to exhibit the highest level of transcription among tpr family genes [80], the fact that *tprK* displays high sequence variability [36] makes the likelihood of detecting this protein minimal due to rigid MS analytical criteria.

**Table 5:** Peptide Sequences of Proteins TprB, C/F/I, E/G/J and H, Detected by MS analysis.

Protein	Peptide Sequence Identified
Tpr protein B	NELAAQMR
Tpr protein B	VKGKGTNSR
Tpr protein C	APMNALNIDALLRMQWK
Tpr protein C/F/I	GEARSGVWAQLQLK
Tpr protein C/F/I	SGVWAQLQLK
Tpr protein C/F/I	TALLWGVGGR
Tpr protein E	TNGTQVVNIDVTVPVNVRQSPVR
Tpr protein E	VEQAVQENIR
Tpr protein E/G/J	AGISASLIEK
Tpr protein E/G/J	DKLLWNVGGR
Tpr protein E/G/J	IPVQDYGWVKPSVTVHASTNRAHLNAPAAGGAVGATYLTK
Tpr protein E/G/J	TTNTVGVSFPLVMR
Tpr protein G	KKTDALDAGQQIR
Tpr protein G	KTDALDAGQQIR
Tpr protein G	TDALDAGQQIR
Tpr protein H	AGDAYTHLIDGLEAGMDVR
Tpr protein H	RVRSVGTWALLFMSSAAGLCAETR
Tpr protein H	LHTLASTPR
Tpr protein H	RTLLSPSAAVR
Tpr protein H	TKVTPGGPVAYAIAQR
Tpr protein I	FIQMALVK
Tpr protein I	VATDSGDR
Tpr protein J	MVGEALIKQQLSR
Tpr protein J	NNANMQAVGGSLGDTARMVGEALIK
Tpr protein J	NNNGNPLPSGGSSGHIGLPVVGK
Tpr protein J	QDLADLVPMMR

### BAM-complex

Outer membrane beta-barrel proteins (OMPs) are commonly involved in cellular process such as small molecule efflux (such as antibiotics) and nutrient acquisition [81,82] in bacteria. The beta-barrel assembly machinery (BAM) complex [83] is thought to facilitate OMP assembly, insertion and folding and in Gram-negative bacteria this complex is typically composed of five proteins: BamA, which is an integral membrane protein and four accessory lipoproteins, BamB-BamE [84]. The insertion and assembly of proteins into the outer membrane is controlled through interactions with periplasmic chaperones (SurA, Skp, and DegP) [85]. Studies [72,86] have demonstrated the presence of a BAM complex in *T*. *pallidum* which is similar to that of *Escherichia coli* [72]. We detected the BamA orthologue (TP0326) [72,87,86,74]. Peptides identified corresponded to the POTRA 2 & 3 domains and a transmembrane domain/ extra-cellular Loop L3 [72,86] (Table 6).

**Table 6:** Peptides Related to BamA orthologue protein (TP0326) identified in MS analysis and corresponding (topological) domain locations.

Identified peptide sequence	Sequence location	(Topological) Domain Location*
MKVDQESLR	141–149	POTRA 2
VDQESLRR	143–150	POTRA 2
AFTESVLK	197–203	POTRA 3
KVLSTQEAR	205–213	POTRA 3
VEGVAKTVDK	246–255	POTRA 3
AGSYGNGLPHPYTSR	514–529	Trans-membrane beta-strand 5/ Extra-cellular Loop L3

*based on experimental predictions [72,86]

### Other detected proteins implicated in *T*. *pallidum* virulence

In our analyses we detected a selection of additional proteins that have been previously implicated in *T*. *pallidum* virulence and pathogenesis, as reported in Table 7.

**Table 7:** Proteins identified in this study that have been previously implicated in *T*. *pallidum* virulence.

ORF Number(s)	Reported/predicted functional role	Supporting reference
TP0967, TP0968, TP0969	TolC-like proteins	[88,89]
TP0155	Fibronectin binding protein	[75]
TP1038	Bacterioferrin/ TpF1	[90]
TP0027, TP0649	Putative hemolysins	[91]

### Exploring the undetected *T*. *pallidum* proteins

Of the predicted protein coding ORFs, 482/968 proteins were not detected in this study. Most of the undetected proteins are classified as ‘uncharacterized proteins/hypothetical proteins’ (N = 197), ‘conserved hypothetical integral membrane proteins (N = 10), or ‘conserved hypothetical protein’ (N = 1). The most plausible explanations for not detecting half of the proteome are i) very low protein abundance could evade MS detection, ii) lack of protein expression during *in vivo* expression during some or all stages of infection, iii) small proteins are less viable to detection since they contain fewer peptides and/or these protein sequences lack arginine or lysine tryptic digestion sites or iv) the presence of (partial) sequence heterogeneity that would thwart peptide/database matching. Certain caveats of MS analyses will always preclude the detection of the whole proteome of organisms. A non-exhaustive list of other technical limitations include: i) hydrophobic peptides do not elute from LC columns during the applied gradient, ii) spectral masking of low abundant proteins by the presence of high abundant protein spectra, iii) co-elution and ion suppression that may prevent the ionization or detectability of some peptides by MS and iv) some peptides are unable to ionize sufficiently on the MS platform.

### Variable *T*. *pallidum* genomic sequences as modulators of protein expression

To address the possibility that the presence of variable sequences may have affected proteome coverage, either by altered gene expression or by precluding MS detection, we searched for known and predicted heterogeneous sequences in the *T*. *pallidum* genome. Within this analysis we looked for sequences containing elements indicative of phase variation (homopolymeric tracts) or antigenic variation through gene conversion (tandem repeats, *tprK* donor sites and quadruplex forming G-rich sequences (G4FS)). Previous investigations have identified and characterized 19 genes with variable sequence elements, of which 9 proteins were detected in this analysis. Aside from the 12 aforementioned Tpr family proteins there are seven additional genes shown to contain variable sequence elements including: *tprK* donor sequences to promote gene conversion (*tp0130; tp0129; tp0128*), homopolymeric G-tracts (poly-G tracts) in promoter regions to alter transcription (TP0126), poly-G tracts in the ORF to induce phase variation (TP0127), or G4FS *cis-acting* DNA elements that form guanine quadruplexes to induce recombination and gene conversion (TP0104; TP0136) [32,36,67,70,92]. Notably, TP0136, a fibronectin binding protein implicated in *T*. *pallidum* virulence not detected in this analysis, harbors two G4FS sequences localized within tandem repeats in the ORF [93]. Surprisingly, the paralogues of TP0136: TP0133, TP0134, TP0462 and TP0463 were also not detected. Among these seven additional variable sequences only an OmpW homologue (TP0126) was detected in our analyses.

We also searched for predicted variable sequences in the *T*. *pallidum* genome. A previous *T*. *pallidum* genomic study predicted the presence of G4FS which may be involved in generation of *tprK* variants in pathogenic treponemes [32]. Similar G4 DNA structures have been implicated in the host immune evasion tactics of *Neisseria gonorrhoeae* where they function as recombination activation elements to regulate gene conversion and the expression of cell surface pilin proteins (PilE) [94]. Giacani *et al*. (2012) identified 46 putative G4FS sequences located in 33 different genes and eight unique intergenic regions (IGRs) of *T*. *pallidum*; 21 of the 33 predicted G4FS-containing ORFs were detected in this analysis. Among the eight putative G4FS residing within unique IGRs, only two of the downstream proteins were detected in this study (TP0104; TP0549). Additionally, we searched for the presence of tandem repeats [95,96] in ORFs and IGRs of genes for which peptides had previously been detected using MS [16] or exhibited high transcript abundance [15]. The presence of highly mutable sites, or contingency loci, such as tandem repeats have been suggested to represent a mechanism for rapid environmental adaptation and virulence within a host [97]. This has been explored in a recent study involving serial *in vivo* passage of *Campylobacter jejuni* in mice that resulted in increased phases in the contingency loci and virulence [98]. This analysis identified three additional genes harboring tandem repeats (*tp0470; tp0424; tp0769*), providing a possible rationale for why these proteins remained undetected in this study. Our study detected 30 proteins out of a total of 60 proteins with known and predicted variable sequences. Remarkably, four proteins discovered in this analysis were only annotated in the original *T*. *pallidum* genome [13], mostly due to the fact that sequences below 150 base pairs were not annotated as protein coding in the resequenced genome [14]. Perhaps there is a need for deeper mining of the *T*. *pallidum* genome and re-evaluation of the definition of protein coding sequences, especially in light of the recent attention brought to classes of endogenous polypeptides called ‘SEPs’ (sORF-encoded polypeptides). These polypeptides are encoded by short open reading frames (small ORFs or smORFs) (generally <150 amino acids in length) in bacteria and eukaryotic organisms and are thought to play an important function in biological functions [99] such as cell survival under conditions of glucose toxicity as studied in *E*. *coli* [100]. Interestingly, in *M*. *pneumonia*, 53% of all smORFs are deemed essential to cell survival whilst another 11% affect the fitness of the organism [101], indicating that these may also play a large (unknown) role in *T*. *pallidum* biological function.

In general, proteins in small genomes are more likely to function as proficient “multitaskers” and have been shown to interact with other proteins from a wider range of functions in comparison to their orthologues in larger genomes [102]. It is possible that many *T*. *pallidum* proteins perform multiple biological functions, especially under different environmental conditions. A growing area in proteomics is the concept of ‘protein moonlighting’, defined as a single protein that displays multiple functions that are not related to multiple RNA splice variants, multiple proteolytic fragments or gene fusions [103]. Many bacterial species employ protein moonlighting and the role of this phenomenon in bacteria virulence has been excellently reviewed by Henderson *et al*. [104,105]. Some bioinformatic approaches have been suggested to approach genome wide annotation of potential moonlighting proteins [106,107]; these may be useful for future *T*. *pallidum* proteome studies.

One of the many intriguing aspects of *T*. *pallidum* is the fact so many proteins lack homology with proteins from other bacteria. This is exemplified by the fact that only 59% (581/968) of the *T*. *pallidum* protein coding genes were allocated into COG or NOG categories. With the demonstrated expression of 114 uncharacterized/ hypothetical *T*. *pallidum* proteins in this study, some even at high abundance, further experimental analysis is needed to elucidate the functions of these proteins such as looking at protein binding partners. Periodic re-evaluations of ‘uncharacterized’ *T*. *pallidum* proteins are warranted, especially with the rapid sophistication of bioinformatics tools and the growing repertoire of proteins with known predicted functions from other organisms.

We are confident in the quality and extent of the protein coverage of this analysis. For example, we performed analysis on three biological replicates, employed multidimensional peptide separation techniques together with complementary MS analyses in order to improve the dynamic range and coverage of the analyses. Nevertheless, there are a number of limitations related to this study.

### Limitations

Even though our experimental approaches aimed to closely mimic the physiological conditions of human infection, a distinct advantage over the artificial conditions of *in vitro* studies, we cannot exclude the effects of inter-rabbit variability. Different rabbits may exert unique immune pressures, which in turn may influence gene expression. The fact that infected rabbits typically do not transition into the secondary stage of syphilis [108] and there is no tertiary stage in rabbits [109] may suggest that the infectious dynamics of rabbit syphilis may differ from that of humans. Moreover, there may be differential gene expression depending upon the tissue environment [15], therefore the analysis of intradermal rather than intratesticular infections of rabbits, or sampling of human syphilitic lesions (pending ethical consent) could provide interesting insights into differing protein expression profiles. Lastly, technical handling after testicular extraction and treponemal purification may ‘stress’ the bacteria into a non-characteristic infectious expression state and some proteins may degrade quickly after extraction since individual protein half-life ranges can vary from several seconds to tens of hours [110]. Gentle and prompt sampling and handling of treponemal extract samples may help to alleviate these potential interferences.

Despite purification efforts through Percoll density gradient centrifugation, the high abundant rabbit albumin may have masked the spectra of some lower abundant *T*. *pallidum* proteins. Additional purification or pre-fractionalization steps could be added to facilitate the detection of low abundant proteins, however, there is a risk of inadvertently depleting treponemal proteins through methods such as albumin depletion. Possible experimental method improvements include altering the LC-MS/MS settings to be focused on either small or large proteins and/or using alternative protease and/or multi protease protein digestion [111]. Regarding the use of spectral counting, this method remains a semi-quantitative estimation of protein abundance since proteins are not measured compared to a reference. More absolute and precise protein quantification methods could be used in the future such as isobaric tags for relative and absolute quantification (iTRAQ) or selected reaction monitoring (SRM) as reviewed by Maaß and Becher [112].

### Conclusions

This study makes a number of contributions to the characterization of the *T*. *pallidum* proteome: i) we detected 557 *T*. *pallidum* proteins expressed during *in vivo* experimental rabbit infection using complementary mass spectrometry detection techniques; this is the first account of 499 proteins at the protein level using these methods, ii) protein abundance semi-quantified by spectral counting showed a low correlation with previous gene expression transcriptome data, iii) 116 predicted membrane localized proteins were detected, of which 16 have evidence supporting outer membrane localization and iv) a number of virulence factors were detected, including 8/12 Tpr proteins.

## Supporting Information

S1 Table Mass spectrometry data reports for biological (N = 3) and technical runs (N = 2). (XLSX)

S2 Table Extensive descriptions of all unique *T*. *pallidum* proteins identified in this study by mass spectrometry analysis. (XLSX)

S3 Table Peptide identifications per biological (N = 3) and MS run (N = 2) and corresponding calculated spectral counting NSAF values. (XLSX)

S4 Table *T*. *pallidum* membrane protein bioinformatic pipeline prediction analyses. (XLSX)
